# Object Recognition and Positioning with Neural Networks: Single Ultrasonic Sensor Scanning Approach

**DOI:** 10.3390/s25041086

**Published:** 2025-02-11

**Authors:** Ahmet Karagoz, Gokhan Dindis

**Affiliations:** Department of Electrical and Electronics Engineering, Faculty of Engineering and Architecture, Eskisehir Osmangazi University, Eskisehir 26040, Türkiye; gdindis@ogu.edu.tr

**Keywords:** ultrasonic sensors, signal classification, convolutional neural networks, signal processing, object recognition, machine learning

## Abstract

Ultrasonic sensing may become a useful technique for distance measurement and object detection when optical visibility is not available. However, the research on detecting multiple target objects and locating their coordinates is limited. This makes it a valuable topic. Reflection signal data obtained from a single ultrasonic sensor may be just enough for the measurements of distance and reflection strength. On the other hand, if extracted properly, a scanned set of signal data by the same sensor holds a significant amount of information about the surrounding geometries. Evaluating this dataset from a single sensor scanning can be a perfect application for convolutional neural networks (CNNs). This study proposes an imaging technique based on a scanned dataset obtained by a single low-cost ultrasonic sensor. So that images are suitable for desired outputs in a CNN, a 3D printer is converted to an ultrasonic image scanner and automated to perform as a data acquisition system for the desired datasets. A deep learning model demonstrated by this work extracts object features using convolutional neural networks (CNNs) and performs coordinate estimation using regression layers. With the proposed solution, by training a reasonable amount of obtained data, 90% accuracy was achieved in the classification and position estimation of multiple objects with the CNN algorithm as a result of converting the signals obtained from ultrasonic sensors into images.

## 1. Introduction

Object recognition and localization play a critical role in many applications, from industrial robotics to autonomous vehicles, and from security systems to logistics management. Ultrasonic sensors are widely used in these areas due to their low cost, reliability and wide detection range.

There are many studies in the literature using cameras in object classification and detection [1]. The optical systems used in computer vision algorithms used for object recognition are sensitive to the amount of light in the air. This directly affects the object recognition process in real-life applications. The use of data obtained from ultrasonic sensors for object recognition has an important place in the literature. The use of these systems for object recognition and positioning in places where camera systems cannot be used is an important research area. Object recognition systems performed with ultrasonic sensors are known for their advantages in areas such as cost-effectiveness, ability to work in dark environments, low energy consumption and ease of integration compared with optical systems [2].

Ultrasonic sensors are used as an effective technology in various applications such as environment sensing, object classification and material recognition. These sensors provide information about both static and dynamic objects by processing time, frequency and time–frequency features obtained from echo signals [3,4,5]. Related studies have focused on various applications, covering a wide range of classification from simple shapes to complex structural obstacles, different types of materials and surface conditions [6,7,8]. Various signal processing techniques such as Hilbert transform, continuous wavelet transform (CWT), fast Fourier transform (FFT) and empirical mode decomposition (EMD) have been used for the analysis of echo signals [5,9,10]. These techniques have supported the classification and recognition processes by enabling the extraction of features such as phase, amplitude, delay and frequency from the signals [11,12,13]. These data have been processed with machine learning algorithms (e.g., CNN, SVM, KNN, decision trees) and have provided high accuracy in tasks such as human detection, material detection and ground type classification [14,15,16]. Methods inspired by biological systems increase the performance of ultrasonic sensors. In particular, they have been inspired by the echolocation mechanisms of animals such as bats and dolphins [8,17,18]. These approaches have optimized environmental sensing and material recognition processes in robotic applications and have been effective in difficult environmental conditions (dust, darkness, environments full of toxic gases) [7,19,20]. As a result, ultrasonic sensors offer an important solution in environmental sensing and classification tasks in autonomous systems, robotic applications and industrial processes with their low-cost, energy-efficient and durable features [21,22,23].

With the proposed method, data acquisition processes are performed by scanning multiple objects with a single ultrasonic sensor integrated into the 3D printer, and the obtained signals are converted into images. The images are given as input to the proposed CNN-based deep learning model, and, in addition to the classification of objects, position estimation is made, unlike studies in the literature. The CNN-based multi-output model proposed for object detection and recognition with ultrasonic sensors has the ability to simultaneously estimate object types (A, B, C) or multiple object types and location information (X, Y coordinates) with high accuracy.

The contributions of this paper, which aims to provide an end-to-end solution to the problem of multiple object classification and location estimation with ultrasound-based sensors using a CNN-based deep learning model, and the motivation for our work are as follows.

The main difference of the proposed method compared with the studies in the literature is that errors are minimized by collecting data with a single ultrasonic sensor on multiple objects with single axis scanning. In this way, an end-to-end reliable solution is provided for the recognition of objects and the estimation of their coordinates. Again, unlike the studies in the literature, instead of using the envelope, amplitude, statistical, time, frequency or echo features of the signal, this method stands out by visualizing the signal analyses in a single image, with the integration provided by single axis scanning and performing the classification processes with image processing. As an output of this, it aims to perform multiple object classification, object recognition and coordinate estimation as in ultrasonic.

The proposed method forms the basis of a system that can perform object recognition and coordinate determination operations integrated with human or robotic applications by scanning objects in real time, together with the algorithm and sensor design to be developed within the scope of future studies in environments without visibility.

In the literature, there is no automated data collection system integrated with ultrasonic sensors for multiple objects. With this developed system, it is now possible to produce datasets very quickly for different object types and to test different scenarios on these datasets. The designed ultrasonic sensor circuit is low cost and provides more accurate results than measurements taken with commercially available and unstable ultrasonic sensors. Applications used for object recognition in the literature mainly require different data pre-processing and feature extraction processes. This situation causes loss of time and more mathematical operations.

The introduction part of the article is followed by a literature review (Section 2) including evaluations from different perspectives regarding ultrasonic sensors for object recognition and detection. The automated data collection system, generated dataset, designed sensor, proposed method and the data pre-processing process are mentioned in the material and methods (Section 3) title. The proposed CNN deep learning model is explained in detail in Section 4. The study is followed by experimental results and discussion (Section 5) and conclusions (Section 6).

## 2. Related Work

A review of the literature reveals that object detection and recognition using ultrasonic sensors remains a significant area of research, with numerous studies conducted in this domain. Simone et al. investigate the use of ultrasonic sensors for dynamic obstacle avoidance. The system aims to increase agricultural precision by retrofitting existing agricultural machinery for autonomous functionality [3]. Pöpperli et al. present an effective method for object height classification using low-cost automotive ultrasonic sensors. The proposed capsule neural network architecture exhibits improved performance over traditional CNNs for automotive detection tasks by achieving high accuracy (99%) and low runtime (0.2 ms) [11]. Shi et al. propose a CNN-based method for automatic classification of ultrasonic signals, focusing on defect detection in mixed stainless steel welds. Higher accuracy is achieved compared with manual methods and traditional feature extraction [6]. Meng et al. apply deep CNNs to classify ultrasonic signals from composite materials. The approach achieves high accuracy in defect detection and visualization via C-scan imaging by integrating wavelet transform features and deep learning [9]. Bystrov et al. investigate the use of ultrasonic sensors to classify road surfaces under various environmental conditions. They emphasize the extraction of signal features for segmentation and apply neural networks for reliable classification, even in difficult terrains such as gravel and soil [17]. This study compares object shape recognition methods using ultrasonic sensor arrays and neural networks. It emphasizes the integration of sensor arrays with machine learning for robust shape recognition under various conditions [19]. Hwasser evaluates the performance of machine learning algorithms including CNNs and CapsNet to classify objects using raw ultrasonic sensor data. The study compares input data types and achieves a classification accuracy of 94% for six object classes using CNNs [12]. Sadh and Huber use high-frequency ultrasonic sensors to detect and classify materials, focusing on water quality and material classification. Fourier transforms and machine learning models are used to identify object features [14].

Ohtani and Baba propose a system using ultrasonic sensor arrays and neural networks for material recycling. The system classifies objects based on shape and material properties without physical contact [21]. Zhang and others apply deep CNNs, including a lightweight architecture called LWTNet, to recognize material textures based on ultrasonic C-scan images [24]. Yan et al. use deep learning techniques to analyze ultrasonic signals to detect cracks in gas pipeline welds. Better accuracy rates are achieved by using the CNN and SVM together, compared with traditional methods [25]. Latete et al. use convolutional neural networks to detect and classify faults in ultrasonic imaging. Data augmentation has been shown to increase the model’s ability to identify flat-bottomed and side-drilled holes in production [26]. This study uses ultrasonic sensors to monitor the structural integrity of wind turbine blades and addresses the challenges encountered in various fault conditions through machine learning and digital signal processing [27]. This paper investigates the use of ultrasonic sensors with CNN and MLP models to classify objects in environments that are not suitable for traditional cameras for object recognition [20]. The study evaluates the importance of phase data for ultrasonic object classification in vehicles. It has been observed that phase features significantly increase the classification rate in complex environments [28]. A scalogram-based signal processing method for ultrasonic detection has been presented and successful results have been achieved with CNN algorithms [22]. Bouhamed et al. propose to use ultrasonic sensing for staircase detection using machine learning techniques considering environmental use for robotic technologies [18]. In this review, the authors discuss the application of neural network-based deep learning to detect acoustic events [29].

Bianco et al. examine the transformative applications of machine learning in acoustics, examining advances in environmental sounds, bioacoustics and source localization [30]. This article discusses material classification using non-contact ultrasonic echo signals for robotic navigation and autonomous vehicle applications. The signal envelope was extracted with the Hilbert transform, and materials such as glass, wood, metal, sponge and fabric were classified with 96% accuracy with a 1-dimensional convolutional neural network (1D-CNN). The work provides high accuracy with low-cost sensors and automatic feature extraction, offering applicability to broader sets of materials in the future [15]. Kroh et al. perform target classification with ultrasonic sonar sensors according to geometric shapes and sizes. In experiments with narrow-band and wide-band signals, artificial neural networks (ANNs) showed over 95% accuracy. They can be used in target geometry identification, navigation and obstacle detection [31]. This study, inspired by the echolocation principles of bats, classified various ground types (grass, concrete, sand, gravel) with over 97% accuracy using ultrasonic sensors. Support vector machines (SVMs) and time–frequency features were analyzed [16]. Kalliris et al. use machine learning algorithms to detect wet surfaces with acoustic measurements. In this study, surface conditions were determined via acoustic echo signals, and high success rates were achieved in wet floor perception [23]. In this study, using an ultrasonic sensor and statistical methods, indoor objects were classified into four classes (edge, flat surface, small cylinder, corner). Linear and Quadratic Discriminant Analysis (LDA/QDA) was applied for feature selection and classification [4].

Another study focuses on identifying humans using ultrasonic sensors with single-class classifiers. A fuzzy-based model distinguished between humans and inanimate objects based on time and frequency features. The results showed higher accuracy compared with the SVM method [7]. Sabatini suggests modeling narrow-band ultrasonic signals with Laguerre polynomials. Objects were classified using echo envelope signals and the robustness of the model to noise was tested [10]. Dror et al. have studied three-dimensional target recognition in different orientations with an echolocation-based neural network model. The effects of time, frequency and time–frequency features were analyzed and spectrogram-based approaches provided the highest accuracy [13]. Ecemis et al. aim to classify objects using spectral information with a sonar-based system. Objects were recognized with 96% accuracy with the Fuzzy ARTMAP neural network. Both frequency and envelope signals were analyzed [5]. In this study, the features obtained by empirical mode decomposition (EMD) were processed with machine learning algorithms such as KNN, SVM and decision trees. The proposed method increased the material detection capabilities of robots in dark, dusty or hazardous environments [8].

The proposed method enables the recognition of objects of varying sizes and distances using a single sensor in zero optic visibility environments, as well as the extraction of their coordinates. As evident from the developed automatic data collection system, the system scans in the single *x*-axis direction and the obtained signals are converted into a single image by the computer. Then, the obtained images are pre-processed and the CNN-based deep learning model, which is customized and optimized by extracting CNN-based features, enables the classification of objects and determination of their positions. First of all, the performance of the system with single axis scanning has been tested with the studies conducted. At the same time, the problems and errors that arise have been analyzed.

In this form, the system constitutes an important step for real-world applications. With the developed system, it will be possible to determine objects in zero-visibility environments in real time by integrating it into a helmet that a person will wear on their head or into robotic systems. For real-time applications, signals can be obtained and processed very quickly sequentially with a powerful local processor integrated into the sensor. Depending on the system it is integrated with, it can be widely used in different areas by performing rotary or spherical scanning as well as single axis scanning.

Table 1 provides a comparative overview of studies on object detection and recognition using ultrasonic signals.

## 3. Materials and Methods

### 3.1. Automated Data Collection System with Ultrasonic Sensor

**Figure 1:**
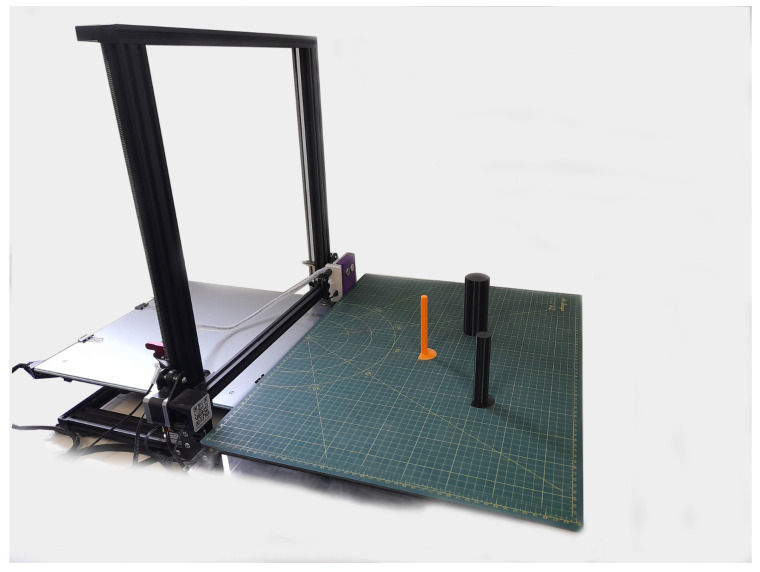
Automated data collection mechanism.

An automated data collection system was built by connecting the Creality CR-10 S4 model 3D printer. All printing heads were replaced with our ultrasonic sensor, thus generating the dataset efficiently and accurately, making a significant contribution to the literature. In this system, a single ultrasonic sensor was mounted in place of the 3D printer, and measurements were taken by scanning in the X direction of the 3D printer with 2 mm steps.The data collection process was recorded by creating 116 different scenarios. This number can be increased for different object types. In this context, the G-code language was used for the 3D printer controller. This code determines the necessary movements and commands for the 3D printer to perform operations in accordance with the coordinates and procedures given. Even though it is cylindrical and spherical, similar results can be obtained with different scanners. Its main purpose is to control the movements and operations of a physical machine. The image of the established mechanism is included in Figure 1.

**Figure 2:**
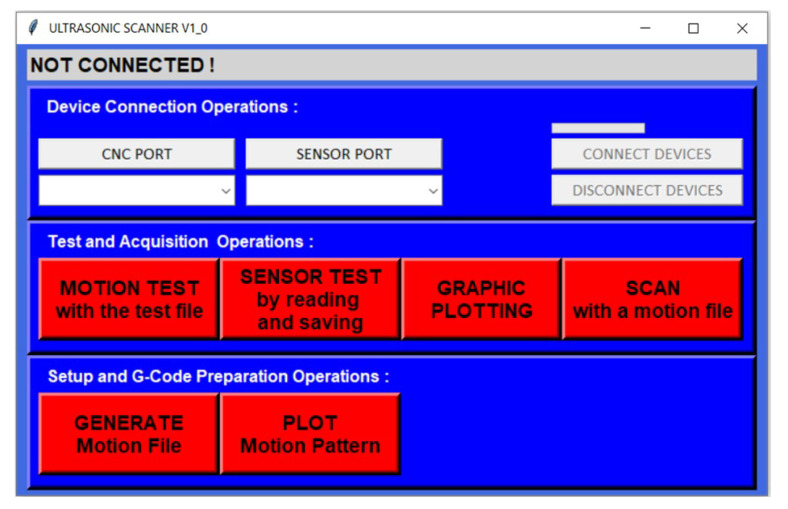
Improved ultrasonic scanner user interface.

For automation, an easily operable graphical user interface (GUI) was prepared in Python 3.9.1. This program includes functions whose features are briefly explained in the buttons in its interface. The screenshot of the developed interface is included in Figure 2.

### 3.2. Dataset

**Figure 3:**
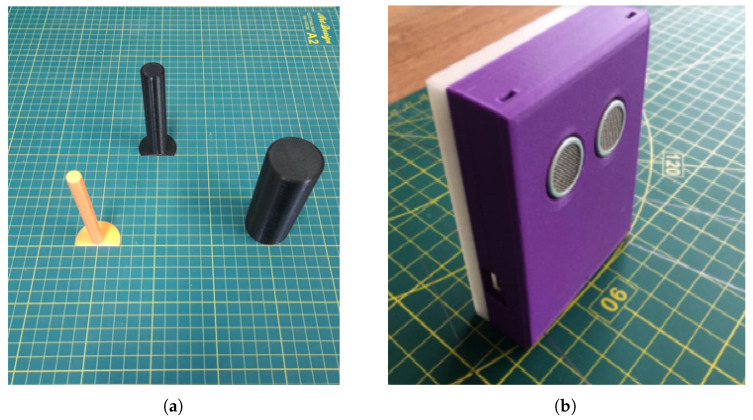
(**a**) Classified objects; (**b**) designed ultrasonic sensor.

This dataset contains 116 labeled cylindrical object images obtained by positioning objects in front of the ultrasonic sensor using one of each, two of each, three of each and mixed up combinations. A maximum 3 objects are used for classification. The details of the 3 classes are as follows: Large-Diameter Object (40 mm), Medium-Diameter Object (20 mm), Narrow-Diameter Object (10 mm). The objects are made of PLA materials printed by 3D printers. The classified objects and the developed ultrasonic sensor are included in Figure 3.

**Figure 4:**
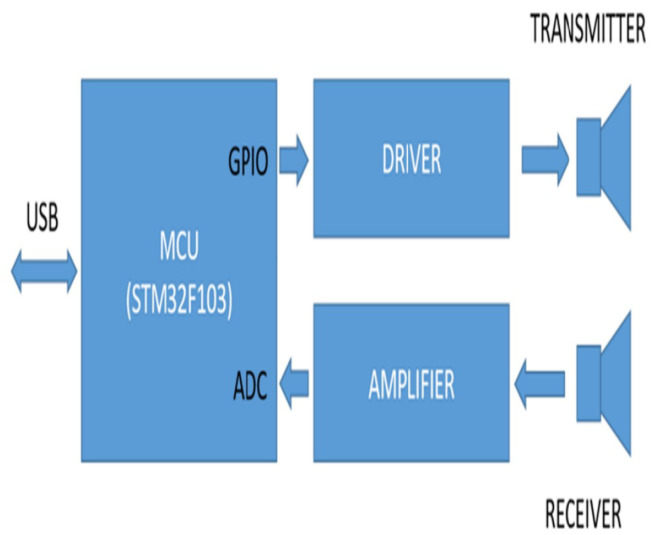
Ultrasonic sensor block diagram.

The ultrasonic data recorder circuit was specifically designed with unique modifications. The SRF04 Sonar Rangefinder (Devantech, Attleborough, UK) module was adapted for data recording purposes by removing the PIC12C508 microcontroller and LP311 comparator chips (Microchip Technology Inc., Chandler, AZ, USA) from its original printed circuit board. Instead, an STM32F103 (STMicroelectronics, Geneva, Switzerland) carrier board was integrated into the design to handle data recording tasks. Ultrasonic transducers used in this research, 400ST/SR160 (Prowave, Queensland, Australia), have been used in our several projects before [32]. They were driven with square wave burst at their 40 KHz (+/−) 1 KHz center resonant frequency. Their main beam angle has 30 degree between their −3 dB points. The STM32F103 microcontroller is well suited for such applications, featuring a 32-bit architecture and capable of operating at a clock speed of up to 72 MHz, offering significantly more computational power compared with the PIC12C508. Additionally, it includes two multi-channel analog-to-digital converter (ADC) modules, each capable of sampling analog signals at a rate of up to 1 mega-sample per second (MSPS). Since the ultrasonic transducers operate at a frequency of 40 kHz, achieving a sampling rate close to 1 MSPS ensures approximately 25 samples per period, providing adequate resolution for the application. Analog input has following specifications: resolution = 12 bit, Vref(−) = 0 V, Vref(+) = 3.3 V, DC offset level of the signal is 1.6 V. A sampling period of 1.66 microseconds was selected, optimized to align with the operating clock frequency and memory constraints, allowing the storage of sufficient data for effective echo distance calculations. This corresponds to 15 samples per cycle of the ultrasonic signal, providing accurate representation. The STM32F103 also features built-in Universal Serial Bus (USB) hardware. By enabling a Virtual Communication Port (VCP), the system facilitates USB communication with a host computer. The unit is powered directly through the USB connection, enhancing simplicity. Once the appropriate firmware was integrated, the entire unit was enclosed in a custom-designed 3D-printed case, making it portable and user-friendly. The interface supports signal acquisition and comparison across four separate channels, allowing for the analysis of signal characteristics from various object types. The recorded data are then collected for use in training neural network. The system includes advanced processing functions, such as subtracting a specific reference signal from the acquired signal to highlight variations, and calculating the envelope of the resulting signal for further analysis. The block diagram of the ultrasonic sensor circuit is given in Figure 4.

### 3.3. Proposed Method Architecture

**Figure 5:**
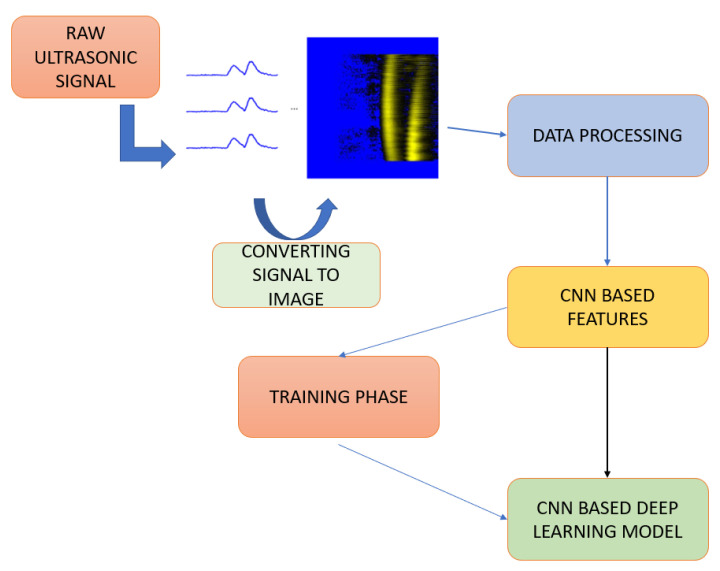
Method architecture flowchart.

The proposed model is a CNN-based deep learning model that provides multiple outputs. The block diagram of the proposed method is given in Figure 5. Additionally, the algorithm of the proposed method has been extracted in detail. See Algorithm 1.
**Algorithm 1** Object Classification and Localization Using Processed Image Data.**Require:** Zipped image dataset $D_{zip}$**Ensure:** Trained multi-output CNN model  1:Extract images from $D_{zip}$ and load as dataset *D*  2:Parse filenames to extract object types and coordinates, storing in DataFrame df  3:Preprocess images in *D* and extract labels, resulting in feature array *X*, type labels $y_{\mathrm{type}}$, and coordinate labels $y_{\mathrm{coords}}$  4:Split *X*, $y_{\mathrm{type}}$, and $y_{\mathrm{coords}}$ into training and testing sets  5:Convert $y_{\mathrm{type}}$ to one-hot encoded labels for each object type  6:Define CNN model with convolutional layers for feature extraction  7:Add classification and coordinate output layers  8:Compile model with loss functions for classification and coordinate regression  9:Train model on training data, using testing data for validation10:**Return** trained model

### 3.4. Data Preprocessing

**Figure 6:**
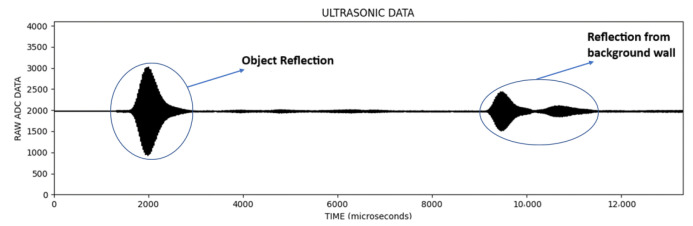
Typical signal information obtained in each scan.

In the signal information obtained from ultrasonic sensors, the vertical axis is the magnitude of the signal and the horizontal axis is the sampling point. An example of the signal data obtained in each scan is given in Figure 6.

**Figure 7:**
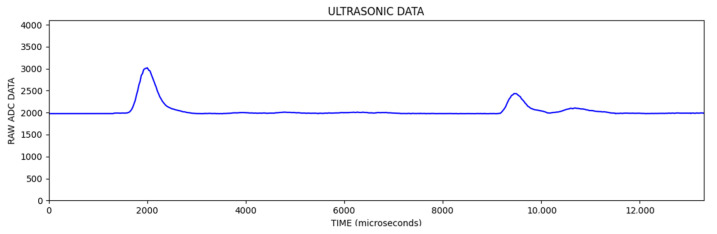
Extracting signal envelope.

In the Figure 6, the first reflection was from the object and the other reflections were from the background wall. In Figure 7, an image line is obtained by extracting the envelope of the signal, which is called an A-Scan, and then converting the vertical value to the pixel color for the corresponding sample (lighter pixel color for the higher vertical value).

**Figure 8:**
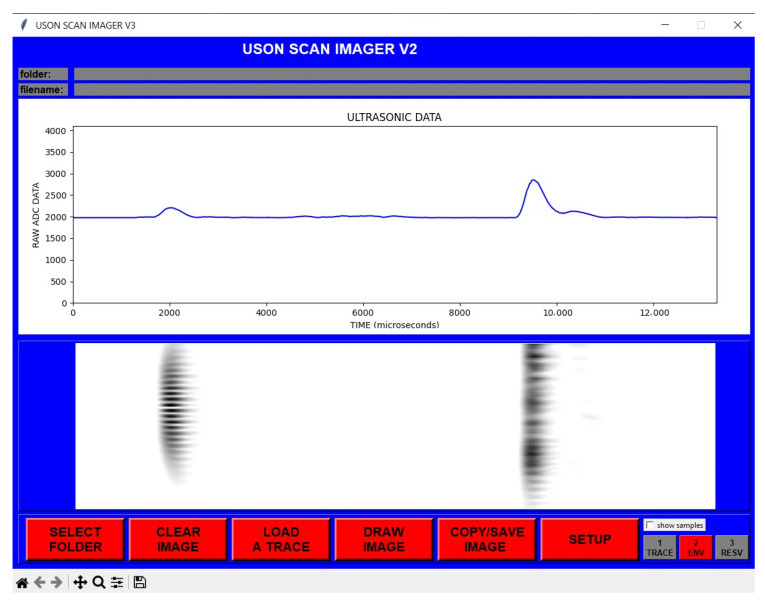
Sample signal information obtained (**top**), and combined with the others to make one image (**bottom**).

When the image lines are combined for each progressive scan, the final image is obtained after all the image lines are combined, as in Figure 8; in the bottom image are what are called C-Scan images in the literature.

The obtained data were divided into separately labeled groups according to object type, distance and coordinate information by the developed software with Python 3.11.5. Following data integration, arrangements were made to read the data in these groups and give them as input to the classification algorithm.

Objects are placed anywhere between 0 and 40 cm on the *x*-axis and in a certain position on the *y*-axis, and while the object is stationary the ultrasonic sensor moves 40 cm on the *x*-axis and takes 201 measurements every 2 mm. These measurements are then converted into images.

**Figure 9:**
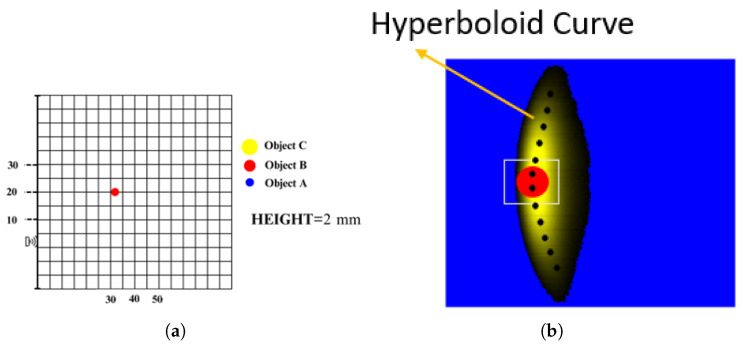
(**a**) Example of object positioning (single object); (**b**) representation of single object on the image.

**Figure 10:**
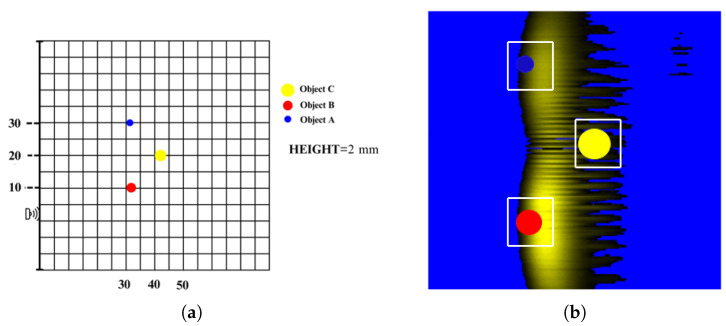
(**a**) Example of object positioning (Object A, Object B and Object C); (**b**) representation of objects on the image (Object A, Object B and Object C).

The reflections from single objects were compared with the reflections from multiple objects. Images obtained from a singular object and multiple objects are shown in Figure 9 and Figure 10, respectively. In images obtained this way, light color regions indicate peaks and dark colors indicate valleys. We should note that images obtained from singular objects have distinct patterns. They are usually in a hyperboloid curve with a continuous color pattern. On the other hand, images obtained from multiple objects have interleaved hyperboloid curve shapes with discrete gaps instead of continuous solid colors.

Within the scope of the study, it is aimed to perform object recognition using a single ultrasonic sensor. However, in the case of multiple object problems, we see that in some signal shots reflections disappear, lose their amplitude where reflections come from the object in different faces and cancel each other. Because of this effect, signal trace with a single sensor depending on location cannot classify the objects correctly and the object recognition success rate is negatively affected.

**Figure 11:**
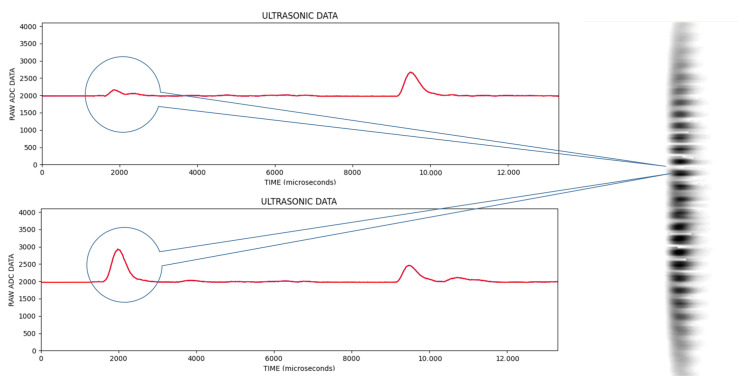
The superposition of the waves by acquired signals from multiple objects. It should be noted that instances are at the 10 mm apart travel distances.

**Figure 12:**
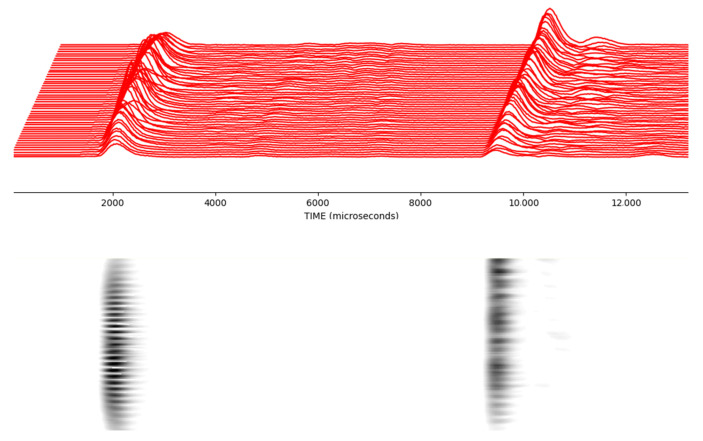
In the process of multiple object recognition, signals obtained as a result of ultrasonic scanning and formation of a single image.

In the upper signal, the trough formed due to the interference of two signals due to the phase difference between the signals reflected from multiple objects is marked. In the lower signal, the peak formed due to the overlapping of signals reflected from multiple objects is marked. This situation reveals that there are limitations in measuring a single signal belonging to objects in the problem of multiple object recognition. Therefore, scanning is good idea to obtain these features so that multiple object recognition processes give a more accurate output. Figure 11 demonstrates that sometimes 10 mm intervals make a big difference in reflection. Using this scanning method, this problem can be converted to a beneficial feature. In order to capture these features more precisely, it is more appropriate to take measurements every 2 mm. If measurements were taken every 5 mm, we would miss some features. As a result of these measurements, each scan is converted into a picture. Instead of manual single measurements, objects are placed anywhere between 0 and 40 cm on the *x*-axis and a certain position on the *y*-axis, and the ultrasonic sensor moves 40 cm on the *x*-axis and takes 201 measurements every 2 mm. Figure 12 shows this much better for each progressive scan.

**Figure 13:**
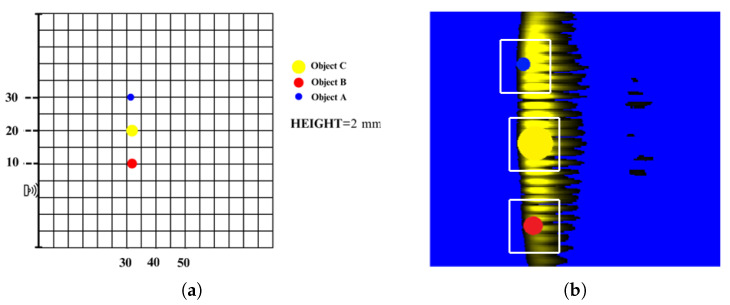
(**a**) Sample location map of objects; (**b**) placement of objects in pictures.

In Figure 13, it is clearly seen that objects create different curved effects on the image depending on their diameters. By taking advantage of these differences, distinctive information can be extracted on the diameters and surface widths of objects.

### 3.5. Normalization Process

The normalization process performed here aims to convert the pixel values of the images from the range of 0 to 255 to the range of 0 to 1. This normalization is performed to enable the neural network to process the inputs better. It prevents high pixel values from excessively affecting the model parameters and allows the model to learn in a more balanced way. It also speeds up the optimization process and facilitates the convergence of the model.

The ultrasonic image data used in the model were obtained compressed in ZIP format. Object types (A = Narrow-Diameter Cylinder (10 mm), B = Medium-Diameter Cylinder (20 mm), C = Large-Diameter Cylinder (40 mm)) and coordinates (X, Y) were extracted from the file names using regex patterns, and this information about each image was converted into a data frame (DataFrame).

Each image was converted to grayscale and normalized according to the input size of the model (128 × 128 pixels). These pre-processing steps optimized the structure of the images to allow the model to predict object types and coordinate information more accurately. Assuming that there are a maximum of three objects in the images, missing objects were filled with the label −1 and coordinates [0, 0].

## 4. The Used Deep Learning Model

### 4.1. CNN Algorithm

**Figure 14:**
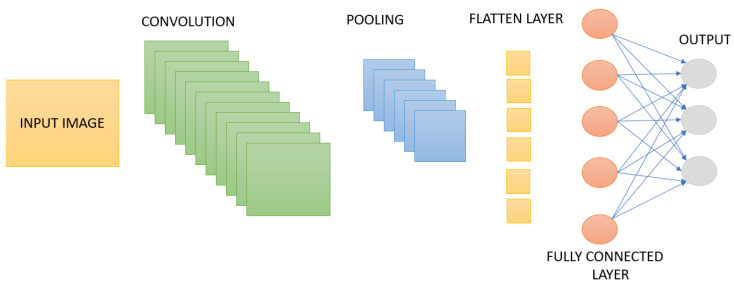
CNN architecture.

Convolutional Neural Networks (CNNs) are a deep learning model that combines fully connected layers with convolutional layers. Mathematically based on the convolution operation in the field of signal processing, CNNs work with the principle of cross-correlation and thus extract important features from data. The basic structure of CNNs consists of two main layers. These are feature extraction and classification layers. While feature extraction is usually performed by convolutional layers and pooling layers, the classification process is performed through fully connected layers. Convolutional layers are not directly connected to each node in the input layer. Instead, they focus on specific regions through small windows called filters or convolutional kernels. This structure allows the network to learn low-level features, and these features are combined to create higher-level patterns. Another important feature of the CNN algorithm is that it can generalize the features it learns in one region to other regions. This is made possible by sharing parameters in the filters and allows the model to produce a similar output at different locations. Pooling layers reduce the computational load and help prevent the overfitting problem of the model. Thus, CNN structures provide a more effective and efficient learning process [6,33]. The general block diagram of the CNN architecture is given in Figure 14.

The CNN model is a pivotal type of neural network that is widely applied in robotics applications for object and target recognition and detection, especially when processing image data [22,30].

Moreover, this algorithm plays an important role in the literature on feature extraction from sound signals and acoustic measurement data in ultrasonics. In essence, the CNN model works as a convolutional neural network layer that extracts features from the input data and transforms them into feature maps. By shifting the kernels along the input, the algorithm calculates the output by multiplying the kernel weights with the input data element-wise. Unlike traditional neural networks, CNNs stand out due to the reduced number of trainable parameters and the faster training process, making them more efficient for complex tasks [34,35,36].

### 4.2. Implementing CNN Model

The model was divided into training sets and test sets to evaluate its generalizability. The training and test sets were divided into 80% training and 20% test ratios.

The Adam optimization algorithm was used in the training of the model, and the categorical_crossentropy loss function was applied for the classification outputs and the mean_squared_error (mse) loss function was applied for the coordinate estimation. This combination is a suitable choice to optimize both the classification accuracy and the coordinate estimation accuracy [37,38].

During the training process of the model, the accuracy and loss values were monitored for 1000 epochs, and techniques such as early stopping were applied to prevent the model from overfitting [37]. Pre-trained weights were not loaded into the model. All layers and weights were randomly initialized and included in the system.

The model was used to classify the images in the test dataset and estimate their coordinates.

The object types predicted by the model were drawn on the image using matplotlib with circle drawings in accordance with the diameter value of the object.

Object types and location estimates are shown in the correct order. Thus, the ability of the model to correctly determine the object type as well as its ability to correctly estimate the coordinates was evaluated.

The classification performance of the model was measured with metrics such as confusion_matrix, accuracy_score, precision, recall and f1_score. The performance, sensitivity and specificity were calculated for each object type and analyzed in more detail [37]. The coordinate estimation performance was analyzed with mse loss. The accuracy rate was evaluated by calculating the mean square error between the estimated values and the real values in the X- and Y-axes for each type of object.

**Figure 15:**
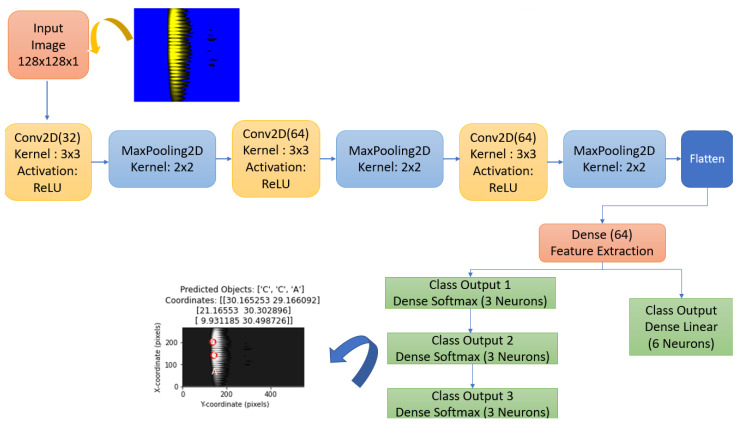
Object recognition-CNN flowchart.

This section details the components of the model. In Figure 15, the flowchart shows the convolution and pooling layers of the model, as well as the classification and coordinate estimation layers. The model produces three separate classification outputs to determine the type of objects. For each object type, the output layers class_output_1, class_output_2 and class_output_3 are created using the Softmax activation function in a Dense layer. This structure allows the model to classify each object independently. A linear regression layer called coord_output is used for coordinate estimation. This layer of the model directly outputs the coordinate values to estimate the positions of the objects on the *x*- and *y*-axes.

A shared convolutional base in the proposed CNN model extracts features for all tasks (classification and regression), improving efficiency and reducing overfitting by leveraging shared information. Images are normalized and resized for consistent input, improving model performance and stability. Predictions are sorted by coordinates, providing important outputs for evaluation and visualization.

The proposed CNN model takes images with 128 × 128 dimensions and 1 channel (black/white) as input to the model. The input dimension represents the height, width and color channels of the image. Convolutional layers allow features such as edges and objects to be extracted from the image. Conv2d layers apply filters by performing convolution on the image. Filters recognize patterns in the image.

The number of filters used in the proposed CNN model is as follows. Layer 1: 32 filters, each with a size of 3 × 3. Layer 2: 64 filters, each with a size of 3 × 3. Layer 3: 64 filters, each with a size of 3 × 3. MaxPooling2d layers take the maximum value in the 2 × 2 matrix to reduce the size of the image. The flatten layer converts the 16 × 16 × 64 feature map into a one-dimensional vector, resulting in a series of 16,384 features.

The model produces four different outputs. Three of them are for object classification and one for coordinate estimation. The goal here is to predict what each object in the image is (A, B, C). In dense layers, the first layer is 64 neurons and is used to learn more features. Output layer: three neurons (three object types). The coordinate output allows the x, y coordinates of three objects in the image to be estimated. The first layer in dense layers is 64 neurons and is again used to learn features. The output layer consists of six neurons for a total of three objects, with x, y coordinates for each object. The activation function is not used in this part because this is a regression, i.e., continuous value estimation problem. Label ordering is performed according to the order of coordinate information on the *x*-axis of the objects in the model’s training dataset from smallest to largest. The first object with the smallest X coordinate in the image is assigned to Class Output 1. The second object is assigned to Class Output 2. The third object with the largest X coordinate is assigned to Class Output 3. If the X values are equal, labeling is performed according to the order of coordinate information on the *y*-axis from smallest to largest.

## 5. Experimental Results and Discussion

**Figure 16:**
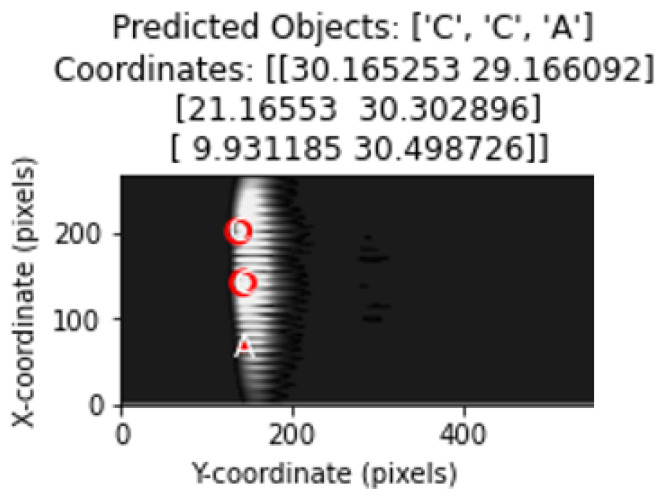
Object and coordinate estimates in test data for 3 objects.

Figure 16 shows representation of the classification and determination of coordinates of objects in a test image containing multiple objects with the proposed method.

Table 2 provides a comparative demonstration of the classification of objects with performance metrics commonly used in the literature in classification algorithms.

The model performs best for OBJECT1. In this class, precision, recall and sensitivity are very high, and false positive and false negative rates are very low. OBJECT2 performs weaker than OBJECT1, but still the performance rates are acceptable. OBJECT3 is the class where the model struggles the most. For this class, both precision and recall are low, indicating that both false positives and false negatives are higher. This indicates that the model has difficulty recognizing objects in less distinct, smaller or complex scenes.

**Figure 17:**
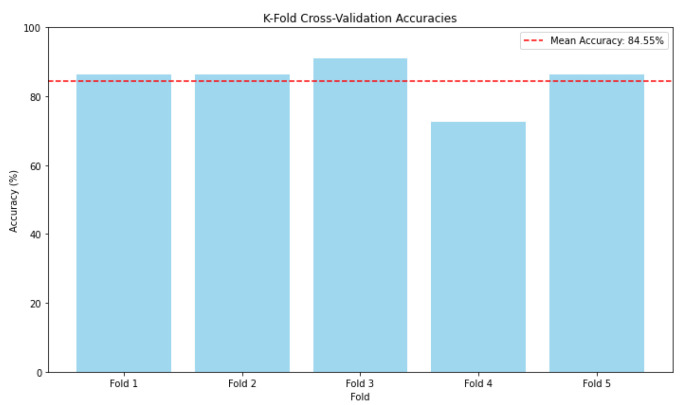
k-fold cross validation accuracies.

Figure 17 shows the k-fold cross validation accuracies across different folds during the evaluation of the proposed CNN model. Each vertical column in the graph represents the model accuracy for a particular fold during cross-validation. The model shows consistent performance across most folds, with an accuracy ranging from approximately 83% to 90%. The dashed red line represents the average accuracy across all folds, which is approximately 84.55%. This provides a general indication of the model performance stability in different data splits. The fourth fold has a significantly lower accuracy compared with the other folds. This can be attributed to the cases of complex images and lack of clarity during multiple object recognition. The model shows stability and relatively high accuracy across most folds, which is a good sign of generalization.

**Figure 18:**
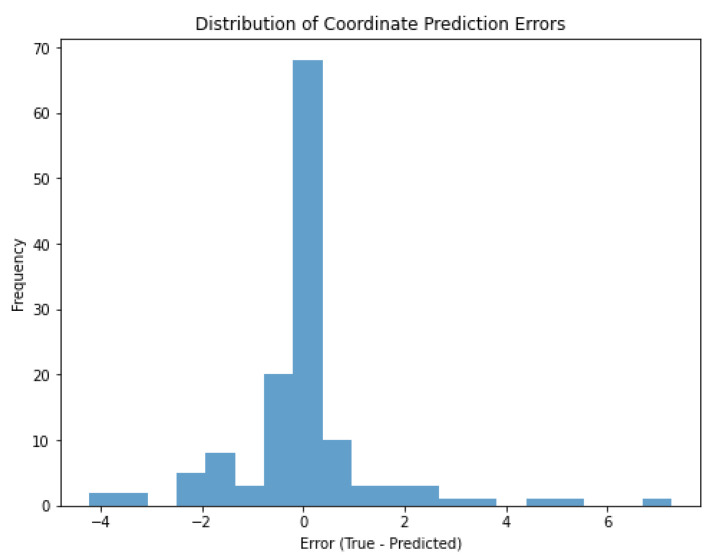
Distribution of coordinate prediction errors in cm.

In Figure 18, a significant portion of the coordinate detection errors are concentrated around 0 cm. This indicates that the estimated coordinates are mostly very close to the true coordinates. The majority of the distribution is between −2 and +2 units (cm). This indicates that the coordinate estimates are generally quite successful and the errors remain within a small range. A few errors beyond ±4 units (cm) were observed. These outliers represent cases where the model has difficulty in estimating the coordinates. For example, these errors were caused by objects being very close to each other, not being able to make a clear separation on the shape, or making classification errors. The high frequency of 0 indicates that many of the model’s estimates match the true coordinates exactly or almost exactly. This is a positive result in terms of model performance in coordinate estimation.

**Figure 19:**
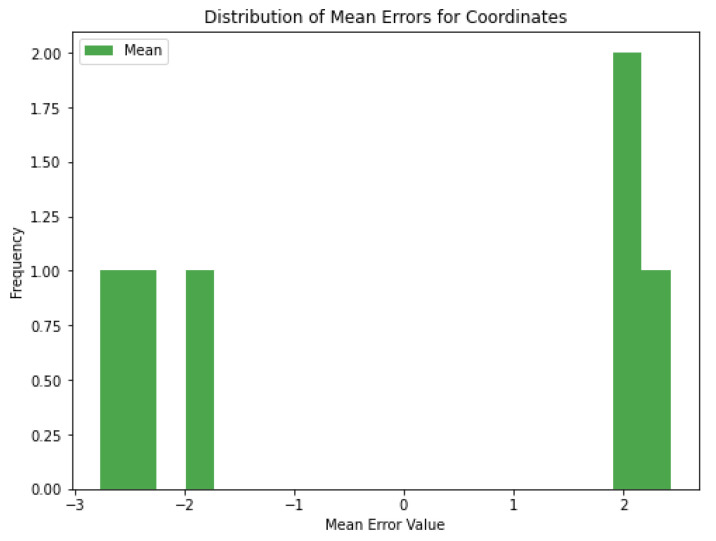
Distribution of mean errors for coordinates in cm.

Figure 19 shows the mean errors for each set of coordinates. For some coordinates, the mean error is positive (e.g., around +2 cm) and for others it is negative (e.g., around −2 cm). The mean errors are generally small, indicating that the model is not making large errors. If the errors are not close to zero, this may indicate a slight bias in the model’s predictions. In some cases, the model can be retrained with more data to reduce the bias. Methods such as adding noise can be further diversified to increase the generalization ability of the model.

**Figure 20:**
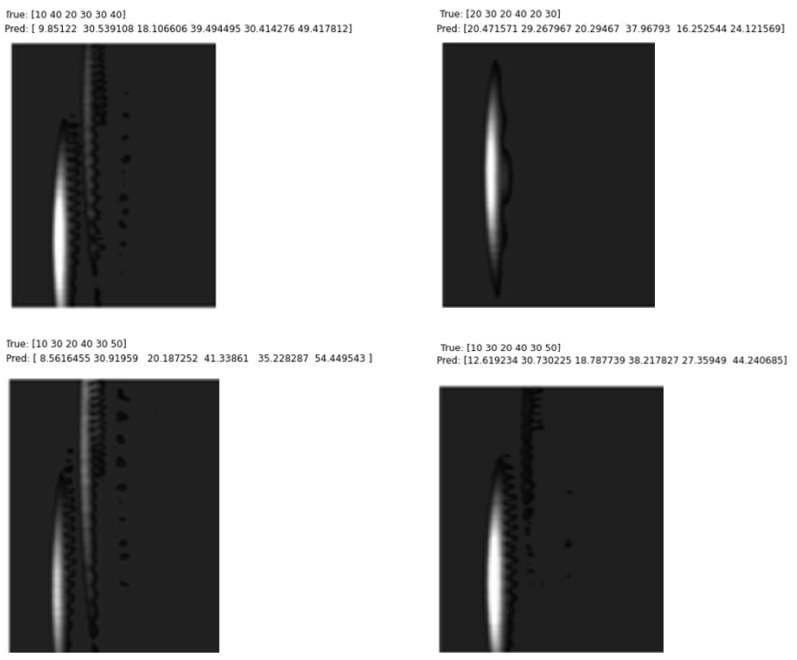
Analysis of images with errors greater than 4 cm.

In order to test the validity of the proposed method, configurations with errors of ±4 cm were analyzed in order to apply the trained NN to difficult scenarios. In the analysis, it was seen that some of the images with coordinate estimation errors of ±4 cm and above and incorrect classification were not clear, which made the detection process difficult. Examples of these images are given in Figure 20.

In order to test the performance of the model close to real-world applications, Gaussian noise and salt-and-pepper noise were separately included in the dataset, and the system performance under noise was tested comparatively. Accordingly, the model is more robust to Gaussian noise. Using data with Gaussian noise added during training can provide the model with more robust performance in real-world conditions. Salt-and-pepper noise degraded the performance of the model more than Gaussian noise. This is because salt-and-pepper noise usually causes sharp contrast changes in the image, making feature extraction difficult. The metrics for the performance of the model in the case of adding Gaussian noise and salt-and-pepper noise are given in Table 3 and Table 4, respectively.

**Figure 21:**
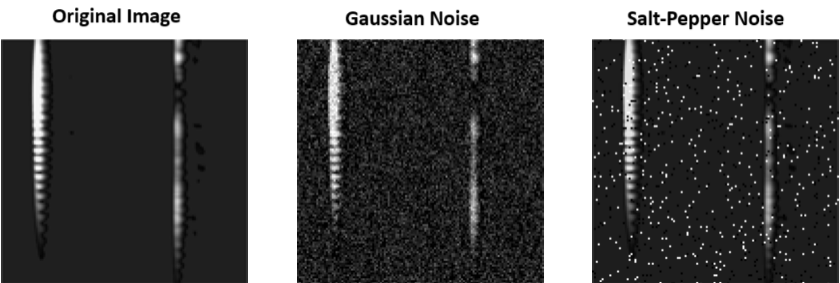
Comparison of original and noisy images.

The comparison of images with Gaussian noise and salt-and-pepper noise added with the original image is shown in Figure 21.

**Figure 22:**
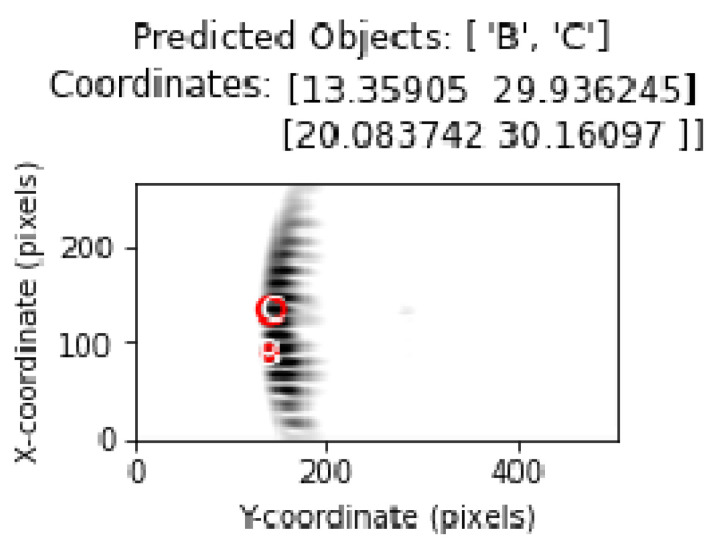
The situation where objects are very close to each other (B (x = 13 cm), C (x = 20 cm)).

**Figure 23:**
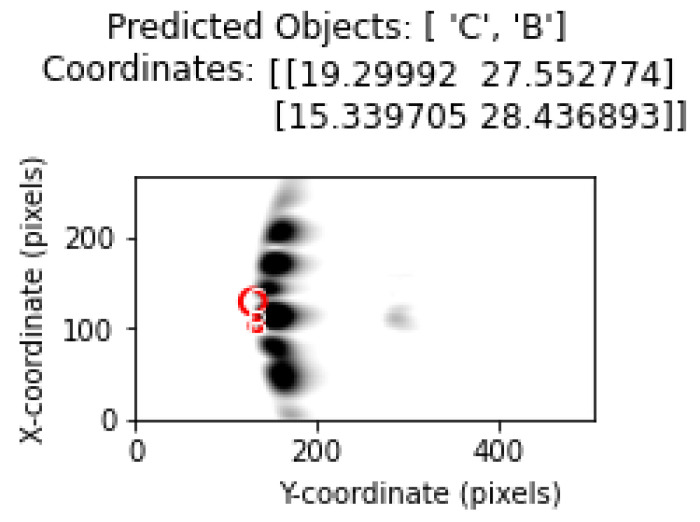
The situation where objects are very close to each other (B (x = 17 cm), C (x = 20 cm)).

By incorporating different analysis methods into the experimental results, important inferences regarding the limitations and performance of the proposed method have been provided. In this section, the output regarding the system behavior in cases where the objects are closest to each other is analyzed. Figure 22 and Figure 23 shows the location and position estimation of objects as a result of classification in images with two objects. In this part of the study, for two objects, measurements were taken at y = 30 cm and the distance between them on the *x*-axis was 3, 5, 7, 9 cm, respectively, and classification studies were carried out. In addition, similar measurements were taken at close range by changing the positions of B and C. The results obtained in Table 5 show that, even in the closest situations, the classification success is at an acceptable level.

**Figure 24:**
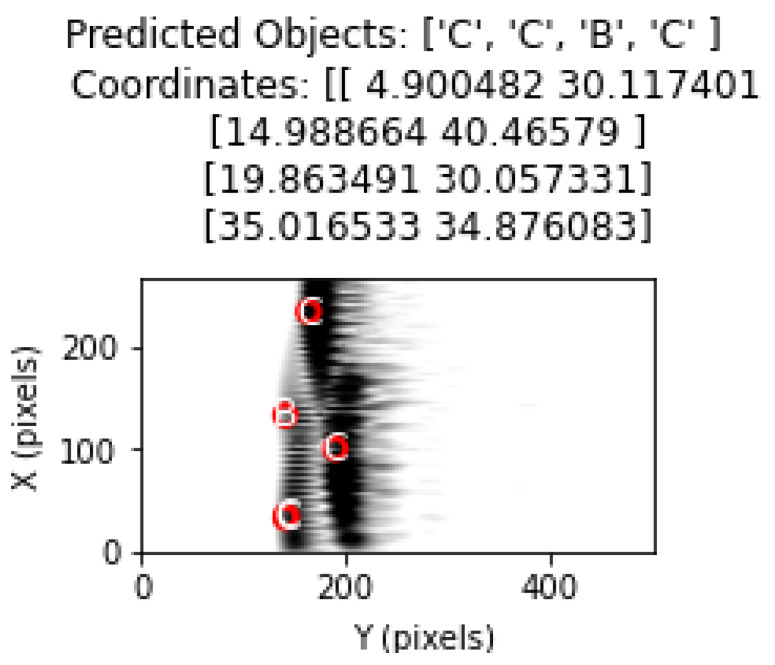
Object and coordinate estimates in test data for 4 objects.

**Figure 25:**
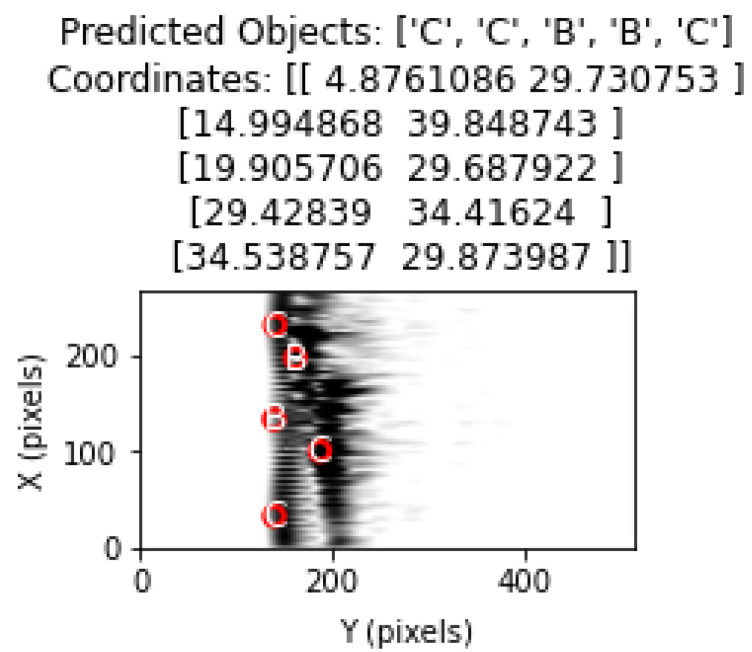
Object and coordinate estimates in test data for 5 objects.

In this section, the performance of the proposed method in cases with four and five objects is analyzed. The classification results obtained in Table 6 and Table 7 for cases with four and five objects show that the proposed method can effectively respond to cases with more than three objects. However, as the complexity increases, the classification performance decreases. Including more cases with four and five objects in the training database will contribute significantly to the classification success. Thus, more information will be extracted from the characteristics of these cases. In the case of four objects in Figure 24, the classification results are quite successful, as in the case of three objects. In Figure 25, the classification performance increased because the B object in the middle was more isolated from the other four objects. The proximity of the B and C objects at the top of the figure had a negative effect on the classification performance. As a result, the system performance and classification success will increase with an effective learning process.

## 6. Conclusions

The study aims to accurately classify multiple objects and determine their coordinates using raw ultrasonic signals of objects of various types and shapes obtained from ultrasonic sensors for autonomous vehicles, robots and other robotic applications. In addition, the object recognition study of the proposed method can be performed with repeated data that can be produced thanks to the automated data acquisition system developed with a single sensor without using a sensor array. The automated data acquisition system built using a 3D printer was a good idea to ensure the validity of the proposed method.

The results achieved, together with the CNN-based model of ultrasonic sensors, are important and can be distinguished from the studies in the literature on multiple object classification and coordinate determination. Within the scope of the study, objects were classified and position estimation was performed with a CNN model that automatically extracts features from input data using three different cylindrical objects and a low-cost sensor.

This study demonstrates both the advantages and limitations of deep learning techniques in extracting object type and location information from multiple-object ultrasonic sensor data. The proposed model was able to predict object types and locations simultaneously with high accuracy.

In the case of multiple object situations, instead of continuous distinct shapes, diffraction patterns are obtained because of the ultrasonic wave length. Discontinuities are observed in the ultrasonic images obtained during multiple object recognition. When these discontinuities are analyzed as a separate parameter, they can serve as valuable information for feature extraction.

Experimental studies have been conducted with different methods in order to reveal the limits and performance of the proposed method in a more effective and understandable way. The images that give the highest coordinate error in the detection of objects have been analyzed. In order to see the situation regarding the system performance in noisy environments, classification has been performed by adding various noises to the dataset. The performance of recognition and coordinate estimation has been examined by taking measurements at the distances where the objects are closest to each other. In addition, the situations experienced in object classification and position estimation in cases where there are four and five objects at the same time have been addressed. The results show that the proposed method has proven itself against the contributions and difficulties provided by the different analysis methods applied. However, there are certainly aspects of the study that need to be improved in order to minimize errors, and increase performance and classification success. More strengthened datasets and different deep learning techniques will constitute the main methodology of our future studies.

In the future studies, the dataset will be expanded, improvements will be made in the image pre-processing section for objects whose coordinates and classes are determined incorrectly, methods for detailed analysis of CNN-based features will be applied, more detailed error analysis will be performed, and comparative evaluations will be carried out with different deep neural network models. At the same time, it is aimed to bring different perspectives to the fore by preparing an experimental setup where rotary and spherical measurements can be made. In real-time applications, scanning can be performed faster by using more than one transmitter and receiver. The system is open to development for different applications and can be improved with a finite number of transmitters and/or receivers.

Furthermore, in environments with zero optic visibility, a real-time imaging system capable of object recognition and coordinate estimation can be developed. This system can be integrated into a helmet worn by a human or robot-integrated, supported by enhanced algorithms and sensor designs.

## Figures and Tables

**Table 1:** Related works.

Ref.	Used Methods	Object Type	Year	Accuracy Rate
[3]	Neural Networks	Cylinder, Cone, Parallelepiped	2018	99
[11]	CapsNet	Object Height Estimation	2019	99
[6]	Neural Networks	Circumferential Composed of Mat.	2022	98
[9]	CNN and SVM	Composite Materials	2017	97
[17]	MDC, ANN-MLP, KNN	Road Surface Classification	2016	96
[19]	ANN	Triangle, Rectangular, Circle	2019	97
[12]	CapsNet and CNN	Triangle, Rectangular, Square, Circle	2020	93
[14]	PCA with NN	Water, Rock, Soap Bar, Sand	2022	95
[21]	NN	Material Identification	2006	99
[24]	CNN	Material Texture Recognition	2020	99.58
[25]	CNN	Gas Pipeline	2020	93.75
[26]	CNN	Phased Array Defects	2021	93.75
[27]	PCA	Wind Turbine Blades	2021	97
[20]	CNN-MLP	Cylinders and Triangular Prisms	2024	93
[28]	CWT-CNN	Bag, Objects, Curb, Tree/Pole, Pedestrian	2024	86
[22]	CWT-CNN	Bag, Objects, Curb, Tree/Pole, Pedestrian	2023	91.5
[18]	SVM	Stair Detection and Recognition	2013	72.41
[29]	CNN, RNN, CRNN	Acoustic Event Detection	2019	–
[30]	Different Algorithms	Acoustic Review	2019	–
[15]	1D-CNN	Glass, Wood, Metal Plate, Sponge, Cloth	2024	96
[31]	ANN	Disc, Cylinder and Hollow Hemisphere	2019	High Rate
[16]	PCA-SVM	Grass, Concrete, Sand, Gravel Terrain Substrates	2018	97
[23]	Decision Tree, SVM, kNN	Road Surface Classification	2019	88
[4]	LDA, QDA	Edge, Plan, Small Cylinder, Corner	2001	High Rate
[7]	SVM	Door, Chair, Glass, Human Being Signal	2015	90
[10]	FLDA	Signal Classification	2001	High Rate
[13]	NN	3D Target Recognition	1995	70
[5]	Fuzzy ARTMAP neural networks	Bottle, Metal Trash Can, Styrofoam Sheet, Lego	1999	90
[8]	kNN, SVM	Material Classification	2023	97.3
Proposed Method	CNN	Cylindrical Objects of Different Diameters	2025	90

**Table 2:** Performance metrics for Object1, Object2 and Object3 results.

Performance Metrics	Object1	Object2	Object3
Accuracy	92%	83%	79%
Precision	93%	82%	79%
Recall	92%	82%	79%
F1 Score	92%	82%	79%
Sensitivity	99%	99%	83%
Specificity	88%	90%	83%

**Table 3:** Performance metrics with Gaussian noise.

Performance Metrics	Object1	Object2	Object3
Accuracy	90%	81%	71%

**Table 4:** Performance metrics with salt-and-pepper noise.

Performance Metrics	Object1	Object2	Object3
Accuracy	76%	79%	70%

**Table 5:** Performance results when objects are very close to each other (B (x = 17 cm), C (x = 20 cm)).

Performance Metrics	Object1	Object2
Accuracy	88%	79%
Precision	90%	84%
Recall	88%	79%
F1 Score	87%	79%

**Table 6:** Performance metrics for four objects.

Performance Metrics	Object1	Object2	Object3	Object4
Accuracy	87%	72%	88%	82%
Precision	83%	71%	88%	79%
Recall	82%	71%	86%	80%
F1 Score	83%	71%	88%	79%

**Table 7:** Performance metrics for five objects.

Performance Metrics	Object1	Object2	Object3	Object4	Object5
Accuracy	84%	73%	88%	68%	75%
Precision	83%	71%	85%	67%	75%
Recall	83%	71%	86%	67%	75%
F1 Score	83%	71%	67%	75%	79%

## Data Availability

Data will be made available by the corresponding author upon reasonable request.
